# Nasal Decolonisation of MRSA

**DOI:** 10.3390/antibiotics8010014

**Published:** 2019-02-04

**Authors:** Peter Mantle

**Affiliations:** Faculty of Natural Sciences, Imperial College London, South Kensington, London SW7 2AZ, UK; p.mantle@imperial.ac.uk

**Keywords:** Mupirocin, urinary biomarker, pseudomonic acid A, monic acid A, MRSA

## Abstract

The recent demonstration for the first time of urinary monic acid A as a clinical urinary biomarker of exposure to intra-nasal mupirocin during medication for methicillin-resistant *Staphylococcus aureus* (MRSA) offers a way of verifying adherence to the regimen. However, absence of the biomarker in some patients needs explanation, to ensure that efficient decolonisation has not been compromised by confounding circumstances, and that additional resistance to mupirocin has not unwittingly been encouraged.

## 1. Introduction

In a context of expected variation in adherence to medication for intra-nasal infection, as is widespread amongst pharmaceuticals, a biomarker for compliance with the mupirocin regimen has been proposed. Demonstration of reliability in a clinical setting has been achieved in a pilot study. However, understanding inconsistencies is necessary through further experiment, not only for nasal decolonization but also to avoid encouragement of mupirocin-resistant pathogens.

Decolonisation of MRSA within nares is a precautionary part of surgical premedication, for which mupirocin (Bactroban; pseudomonic acid A) is widely prescribed [1]. The rapid intra-gastric cleavage to monic acid A, discovered during its development in the 1970s, limited its clinical use to topical application [2]. Notably, mupirocin is not absorbed through skin [1]; thus its intra-nasal function as an antibiotic for MRSA is superficial on nasal membranes. 

Unreliable adherence to regimens for prescribed pharmaceuticals is a well-recognised problem in clinical medicine, to which antibiotics are particularly prone, particularly amongst out-patients. Usually there is no means of monitoring compliance in real time or in retrospect. From clinical experience, a challenge for the first time to devise a method to monitor efficient intra-nasal application of mupirocin was addressed, because its degradation in humans to monic acid A is known and a unique reference sample was available from its original synthesis and subsequent scientific use [2,3]. There was absolutely no precedent for choosing to work with mupirocin on account of any suspicion of its efficacy as an antibiotic, particularly for its relevance in MRSA control. A first step towards assessing the extent of any default was by showing that monic acid A could be extracted from urine, spiked in trace amount, recognised during liquid chromatography/mass spectrometry and identified as a mass-spectral fragment ion [4]. Further exploration as an informal pilot project to demonstrate sensitive methodology for detecting monic acid A in urine during medication needed to be done in a clinical setting. This required ethical compliance, not least because findings might justify publication. This was achieved in a hospital setting with a few catheterised patients under provision of the Imperial College Biobank. Urine samples were collected after the second of the three daily applications of intra-nasal mupirocin and one or two days after. Selective isolation of monic acid A with other organic acids by a Waters solid phase extraction cartridge and eluted metabolites analysed by liquid chromatography-mass spectrometry methodology developed to recognize traces of monic acid A. Monic acid A was selectively isolated from urine of a young adult during medication and consistently recognised quantitatively [5]. 

However, a matter of concern in the recent pilot study is the other two patients, whose urines during mupirocin medication showed no evidence of monic acid A. The significance is unclear but, although elderly and with several morbidities, they were currently residing in an environment of exceptional clinical care. Thus, what factors might limit detection of urinary monic acid A in a hospital context? Could any impair efficient nasal decolonisation of MRSA by mupirocin?

Intra-nasal application of a cream, as mupirocin is formulated, can cause an uncomfortable sensation, in contrast to oral medications with tablets or liquids. There may be dis-inclination to sniff the antibiotic cream fully up and across nasal membranes. An inevitable outcome of comprehensive nasal distribution would be that traces of mupirocin are swallowed; this is harmless but potentially facilitates detection of monic acid A in urine as a biomarker of efficient medication. It is important to reiterate that mupirocin is not absorbed through skin, even that lining the nares. Therefore, cursory application of the cream within nares would not be reflected by detectable monic acid A in urine. A natural reflex to inhale via mouth instead of nose could also appear to have satisfied the need for further distribution. Do nurses who administer routine medication understand the importance of optimising trans-membranal distribution when using mupirocin cream for decolonization of MRSA fully across nasal membranes? Is application always supervised or may a patient sometimes prefer to, or indeed have to, self-medicate? Can patients’ confounding morbidities make administration less efficient? Could mupirocin be made more pleasurable to receive?

There is also concern that variable distribution of mupirocin across nasal membranes may positively encourage selection of mupirocin resistance. Mupirocin resistance has been recognised in MRSA since increasing after a 1990 Canadian hospital epidemic from 2.7 % to 65 % through its 3 year study [6], attributed to increased use of mupirocin ointment as an adjunct to infection control measures. The writer recollects the 1970s and 80s at Imperial College with biochemistry students analysing 3000 L. pilot plant fermentation broth of *Pseudomonas fluorescens* producing pseudomonic acid A, using the classical dilution-plate bioassay with *Staphylococcus aureus.* Occasional bacterial colonies within inhibition zones, implying selection of resistant forms of *S. aureus*, were regularly encountered, even though that culture had never previously been exposed to *S. aureus*. Development of a high-level resistance to mupirocin is of concern [7]. Recent data on antibiotic resistance in skin infections in Greece has shown mupirocin resistance in 12.7 % of total isolates [8]. Anyone doubting the bacterial potential for developing antibiotic resistance should view the admirable online video of *Escherichia coli* while progressing across a thousand-fold antibiotic concentration gradient [9]. Differentiation of current low- and high-level genomic variants among 795 patients in South-East London recognised mupirocin resistance in MRSA isolates, including presumably in patients for whom mupirocin was subsequently administered in hospital [10]. Notably, authors partly attributed the resistance to ‘selection due to mupirocin use’ but did not have data on use of mupirocin for individual patients. However, monitoring the quality of delivering the antibiotic medication by seeking the new urinary monic acid A biomarker might show the real nature of that exposure for individuals, and reveal implied imperfect distribution of the medication across nasal membranes.

Considering a wider context, analytical extrapolation from mass spectral fragmentation characteristics of monic acid A may be valid also for pseudomonic acid B, by taking account of an oxygen atom added to pseudomonic acid A at position 8 though not conveying useful bioactivity. Biosynthetic inter-relationship between pseudomonic acids B and A has been demonstrated both by radiolabelling and molecular genetic methodologies [11,12]. Other compounds with similar structural moieties, such as thiomarinols, also show marked antibiotic activity and have a hydroxyl substituent in a similar position in what may be a potential urinary biomarker. From consideration of its molecular structure, thiomarinol G [13] could yield a degradation product with MS fragment ions similar to those from monic acid A, but the principal MS ions of monic acid A probably remain a urinary diagnostic for efficient adherence to the regimen for nasal decolonisation of MRSA by mupirocin.

Protecting the very particular mode of antibiosis of mupirocin may be easier than discovering new antibiotics. Methodology is now in place [5] to verify adherence to effective mupirocin administration both within hospital and for out-patients. It could also enable more comprehensive experimental clinical study of factors encouraging mupirocin resistance. Additional data to establish the extent of positive biomarker findings under hospital conditions is desirable, as is analytical refinement for monic acid A detection in smaller urine volumes. Study extension to include patients self-medicating in the community may prove even more important in ensuring maximum intra-nasal medication from mupirocin. The writer has already attempted this, in collaboration, under the even more complex ethical compliance regulations, but was frustrated by patient failure to provide the promised sample. Nevertheless, in tackling the global problem of antibiotic resistance, comprehensive study to optimize use of existing resources could now be made, physician-led and with dedicated research resources, while refining the recent methodology for monitoring intra-nasal mupirocin medication.

## 2. Conclusions

Wider discussion of recent demonstration of urinary monic acid A as a biomarker of rigorous intra-nasal medication with mupirocin emphasizes as yet unexplained variation in its expression in a hospital setting. Use of the analytical methodology for further clinical experimentation is encouraged.
